# Antineutrophil Cytoplasmic Antibodies Contribute to Airway Inflammation via Induction of Neutrophil Extracellular Traps in Children With Bronchiolitis Obliterans

**DOI:** 10.1111/crj.70145

**Published:** 2026-01-08

**Authors:** Xiaowen Chen, Shangzhi Wu, Zhenwei Liu, Zhanhang Huang, Jiaxing Xu, Zhongji Wu, Hui Li, Hongwei Li, Dehui Chen

**Affiliations:** ^1^ Department of Pediatrics The First Affiliated Hospital of Guangzhou Medical University Guangzhou Guangdong China

**Keywords:** antineutrophil cytoplasmic, bronchiolitis obliterans, extracellular traps, inflammation, neutrophil

## Abstract

**Objective:**

It was found that the levels of antineutrophil cytoplasmic antibodies (ANCA) are elevated and linked to disease severity of bronchiolitis obliterans (BO) in children. This study aims to explore the mechanism of ANCA in the process of BO.

**Methods:**

Plasma from BO patients (*n* = 40) and healthy controls (*n* = 11) was analyzed for ANCA and neutrophil extracellular traps (NETs) components. Plasma IgG from ANCA‐positive BO children and normal controls were used to stimulate neutrophils, measuring reactive oxygen species (ROS) and NETs production. Small airway epithelial cells (SAECs) were exposed to NETs, assessing viability by CCK8 and cytokine release by ELISA. The IgG treated neutrophils were co‐cultured with SAECs, and cytokines were measured by ELISA.

**Results:**

The levels of ANCA and NETs components including dsDNA, neutrophil elastase (NE) and myeloperoxidase (MPO) in the plasma of BO children were significantly higher than those of healthy controls. ANCA‐positive IgG induced neutrophils produce ROS and NETs. The cell viability of SAECs was significantly reduced upon treatment with NETs in a concentration‐dependent manner. The levels of IL‐8, IL‐17, TNF‐*α*, and TGF‐*β* secreted by SAECs treated with NETs were increased significantly, and the degree of increase was positively correlated with the concentration of NETs. The co‐culture of neutrophils stimulated by ANCA IgG with SAECs significantly increased the expression of cytokines including IL‐8, IL‐17, TNF‐*α*, and TGF‐*β*.

**Conclusions:**

NETs induced by ANCA may exacerbate airway inflammation in children with BO.

## Graphical abstract

This study demonstrates that antineutrophil cytoplasmic antibodies (ANCA) induce neutrophil extracellular traps (NETs) in children with bronchiolitis obliterans (BO). NETs promote airway epithelial inflammation and cytokine release, contributing to chronic airway injury. These findings reveal a novel mechanism by which ANCA may drive inflammatory pathology in BO.

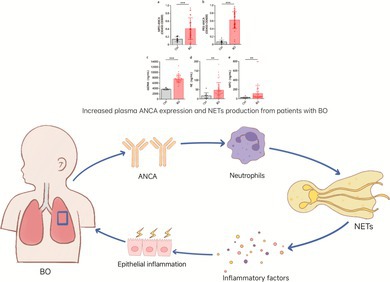

## 1 Introduction

Bronchiolitis obliterans (BO) in children is a chronic lung disease with high disability and mortality, which is characterized by irreversible fibrosis of small airways [1]. The pathogenic cascade involving sustained airway epithelial hyperinflammation and dysregulated repair mechanisms plays a determinant role in BO development [2]. Antineutrophil cytoplasmic antibodies (ANCA) are a series of autoantibodies targeted at the cytoplasmic components of neutrophils, of which the most clinically significant ones are myeloperoxidase (MPO)‐ANCA and proteinase‐3 (PR3)‐ANCA [3]. ANCA has been mostly studied in autoimmune diseases such as ANCA‐associated vasculitis and systemic lupus erythematosus (SLE) [4, 5, 6]. In previous studies, we found significant increases in both MPO‐ANCA and PR3‐ANCA levels in the serum of children with BO [7, 8], with ANCA levels positively correlated with disease severity [9]. Some studies have found that ANCA mediates a series of inflammatory responses via activating polymorphonuclear leukocytes (PMNs; neutrophils) to produce neutrophil extracellular traps (NETs) [10, 11]. NETs are reticular fibrous structures with double‐stranded DNA (dsDNA) as the skeleton and embedded with histones, neutrophil elastase (NE), matrix metalloproteinases‐9 (MMP‐9), myeloperoxidase (MPO) and other cytoplasmic proteins [12]. Notably, ROS serve as the fundamental mediator in the classical NETosis pathway [13]. Under pathological conditions, NETs can directly damage host tissues and cells, or participate in the destruction of host immune tolerance by acting as autoantigens and inducing excessive immune inflammatory responses. The mechanism of ANCA in BO remains unclear, and whether NETs play a role in BO pathogenesis has not been reported. The aim of this study is to explore the pathways of ANCA in children with BO and to clarify whether NETs are involved.

## 2 Materials and Methods

### 2.1 Study Subjects

We recruited 40 pediatric patients with post‐infectious BO (PIBO) and 11 age‐ and sex‐matched healthy children (Table 1) from the First Affiliated Hospital of Guangzhou Medical University, Guangdong, southern China. PIBO was diagnosed during follow‐up in children with a history of severe lower respiratory tract infection who presented with the classical signs and symptoms [14]. All study subjects provided written informed consent at the time of recruitment. The study was approved by the First Affiliated Hospital of Guangzhou Medical University (ES‐2023‐011‐01).

**TABLE 1:** Demographic characteristics of healthy controls and patients with BO.

Characteristics	Healthy donors (*n* = 11)	BO patients (*n* = 40)	*p*
Age, mean ± SD	3.7 ± 0.8	4.6 ± 2.0	0.173
Gender, male/female	6/5	26/14	0.525

### 2.2 Quantification of NETs Markers

We detected dsDNA, NE, and MPO as a measure of NETs in plasma. Levels of dsDNA in plasma from healthy children and BO patients were measured using a fluorometric assay for double‐stranded DNA Quant‐iT PicoGreen dsDNA Assay kit (Invitrogen, USA) following the manufacturer's instructions. Enzyme‐linked immunosorbent assay (ELISA) was performed according to the manufacturer's protocol to quantify the plasma levels of NE (Abcam, UK) and MPO (R&D Systems, USA).

### 2.3 MPO‐ and PR3‐ANCA ELISA Assay

High‐binding 96‐well plates were coated overnight with either 100 ng MPO or PR3 (Sino Biological, Beijing, China) at 4°C. Plates were blocked with 1% BSA (Sigma–Aldrich, USA) at 37°C for 2 h after wash steps with phosphate buffer pH 7.2. Plasma from BO patients at 1/100 dilution was added in duplicates into appropriate microtiter wells and incubated at 25°C for 1 h. Wells were washed and incubated with a 1:5000 dilution of goat anti‐human IgG‐horseradish peroxidase (Sino Biological, Beijing, China) at 25°C for 1 h. After washing, tetramethylbenzidine was used as the substrate at room temperature, and the 96‐well microtiter plate was analyzed spectrophotometrically at 450 and 600 nm after 30 min.

### 2.4 PMNs Isolation

We isolated PMNs from freshly collected whole blood of healthy donors using a Human Neutrophil Isolation Kit (Haoyang, Tianjin, China) according to the manufacturer's instructions. We suspended the PMNs to a concentration of 1 × 10^6^ cells per milliliter in RPMI 1640 supplemented with 10% FBS. Neutrophil purity above 95% was assessed by flow cytometry analysis.

### 2.5 IgG Preparation

IgG was isolated from plasma obtained from ANCA‐positive BO patients or healthy controls (ANCA‐negative) using BeyoMag Protein A + G Magnetic Beads (Beyotime, Shanghai, China) according to the manufacturer's protocol. A total of 21 IgG samples with positive MPO‐ and PR3‐ANCA were used, the positivity of which was confirmed by the previously described antigen‐specific ELISA assay.

### 2.6 Generation of NETs Using ANCA

Neutrophils were resuspended in RPMI 1640 media at a density of 1.8 × 10^6^ cells per milliliter and were stimulated with 5 μg/mL ANCA‐positive IgG or control IgG for 24 h at 37°C. To detect the effect of ANCA inducing neutrophil respiratory burst, PMNs were stained with dihydrorhodamine 123 (DHR123) (Aladdin, Shanghai, China) for 20 min at 37°C in the dark. The intracellular ROS was determined by measuring the absorbance at 488 nm using a fluorescence microplate reader. To isolate and quantify the NETs, the supernatant was discarded. The layer of NETs present at the bottom of the plate was harvested using PBS and collected into a centrifuge tube. After centrifugation at 20 × g for 5 min, the cell‐free NETs‐rich supernatant was obtained. To quantify NETs, we measured free DNA using the PicoGreen dsDNA assay (Invitrogen, USA).

### 2.7 Immunofluorescence Staining for NETs

To stain for NETs, neutrophils were blocked with 1% normal goat serum and incubated overnight with an anti‐MPO antibody (Beyotime, Shanghai, China) at 4°C. For fluorescence immunostaining, goat anti‐rabbit IgG (BOSTER, Wuhan, China) was used as the secondary antibody and incubated for 1 h at room temperature. Coverslips were counterstained with DAPI (Sigma–Aldrich, USA) to visualize nuclear, and analyzed with confocal microscopy (LSM8000, Zeiss, Germany).

### 2.8 Human Airway Epithelial Cells Culture and Treatments With NETs

The human small airway epithelial cells (SAECs) were purchased from Procell Life Science and Technology Company (Wuhan, China) and were cultivated in DMEM high glucose supplemented with 10% FBS, 1% penicillin–streptomycin solution and 1% L‐glutamine (all purchased by Procell, Wuhan, China) at 37°C with 5% CO_2_. SAECs were seeded in 24‐well plates up to 90% confluency, washed once with PBS, and NETs in different concentrations were added. Approximately 5, 10, or 20 μg/mL DNA‐NET, respectively, were added to different wells. The total volume in each well was kept equal by adding DMEM medium. After 24 h, the medium supernatant was collected to perform cytokine quantification as described below.

### 2.9 Co‐Culture of SAECs and PMNs

SAECs were grown to confluence at the bottom of 24‐well Transwell dishes (0.4 μm pore size, Corning Costar, USA). PMNs (5 × 10^6^/mL) were added to the top chambers of Transwell dishes, untreated or preincubated with 5 μg/mL ANCA IgG or control IgG. After 24 h, the medium supernatant was collected to perform cytokine quantification as described below.

### 2.10 Cytokines Quantification

In order to quantify cytokines released in the supernatant of the cell culture medium, ELISA assays were performed. We measured the levels of IL‐8, IL‐17, TNF‐*α*, and TGF‐*β* in equal volumes of different conditional supernatants using the ELISA Kits (Sino Biological, Beijing, China) for IL‐8, IL‐17, TNF‐*α*, and TGF‐*β* respectively, according to the manufacturer's instructions.

### 2.11 CCK‐8 Assay

The cell viability of SAECs was determined by a Cell Counting Kit‐8 (CCK‐8) assay (Beyotime, Shanghai, China). The SAECs treated with different concentrations of NETs were respectively seeded in 96‐well plates and the total volume was made up to 100 μL. Thereafter, 10 μL of CCK8 reagent was added to each well and the absorbance value was measured at 450 nm after 3 h of incubation.

### 2.12 Statistical Analysis

The data was analyzed using SPSS 21.0 software, and GraphPad Prism 6.0 software was used for the graphs. The Mann–Whitney *U* test was used to compare patient demographics and levels of indicators. All in vitro experiments were performed in triplicate, and values were presented as mean ± standard deviation (SD). Statistical differences between in vitro experiments for the groups were determined by a two‐way ANOVA followed by an independent‐samples *t*‐test. Differences were considered statistically significant at *p < 0.05*.

## 3 Results

### 3.1 Increased ANCA Expression and NETs Production in Plasma From Patients With BO

**FIGURE 1:**
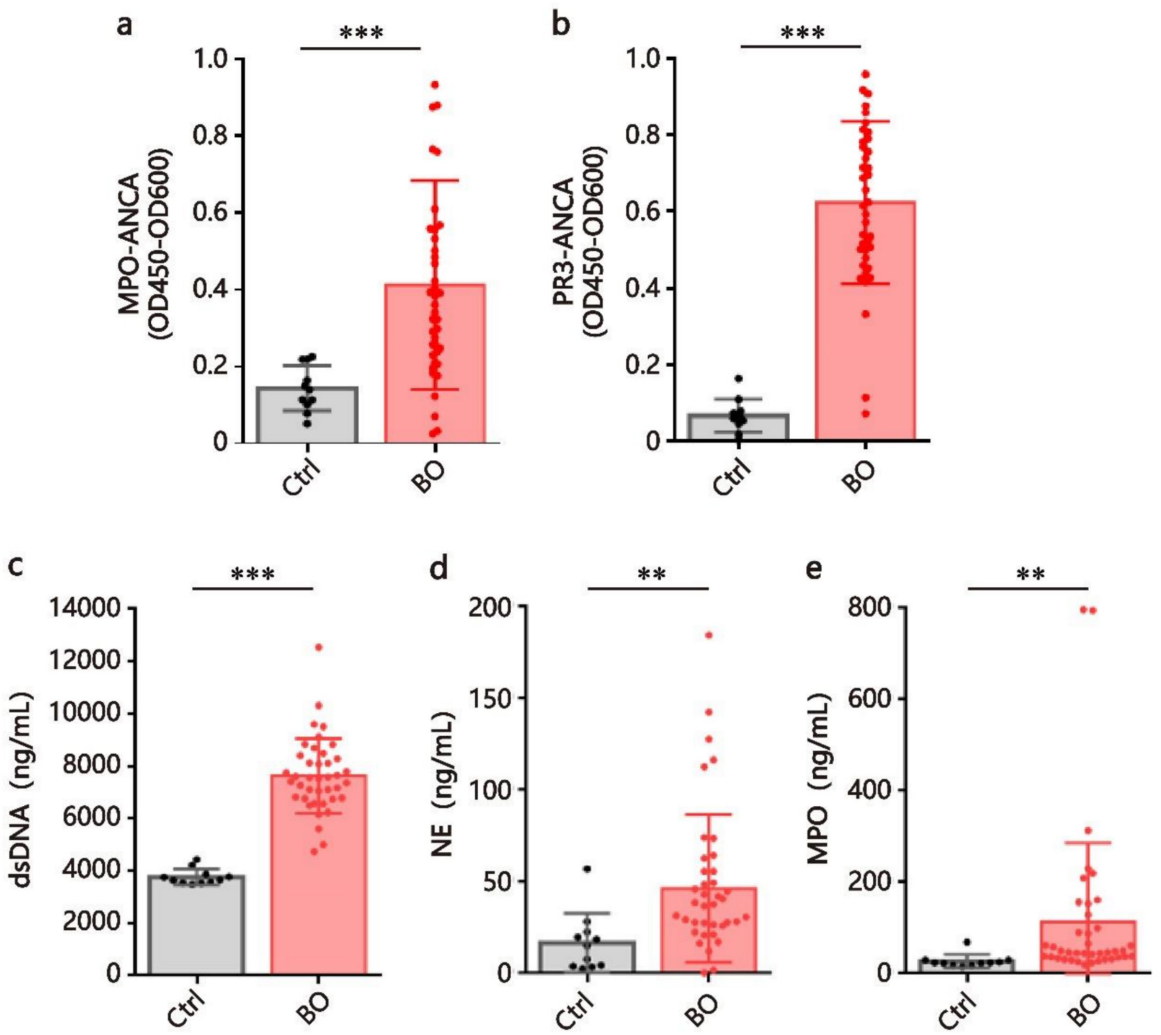
Increased plasma ANCA expression and NETs production from patients with BO. Absorbance of MPO‐ANCA (a), PR3‐ANCA (b), and concentration of dsDNA (c), NE (d), and MPO (e) in plasma of age‐ and sex‐matched healthy controls (*n* = 11), and patients with BO (*n* = 40). Each colored dot represents an individual participant. Data are represented as mean ± SD **p < 0.01, ***p < 0.001. Ctrl, control group.

We assessed the expression of ANCA in plasma from 40 BO patients and 11 age‐ and sex‐matched healthy donors. ELISA revealed a significant increase of both MPO‐ANCA and PR3‐ANCA in BO patients compared with healthy donors (*p* < 0.001 for both; Figure 1a,b), confirming our previous studies. Next, we determined whether NETs production was also elevated in BO patients. We found that plasma levels of NETs markers including dsDNA, NE, and MPO were significantly higher in the BO group than in the control group (*p* < 0.001, *p* = 0.001, and *p* = 0.003, respectively; Figure 1c–e), demonstrating that ANCA, as well as NETs, are highly expressed in the circulation during the process of BO.

### 3.2 Induction of NETs by ANCA in Peripheral Blood PMNs

**FIGURE 2:**
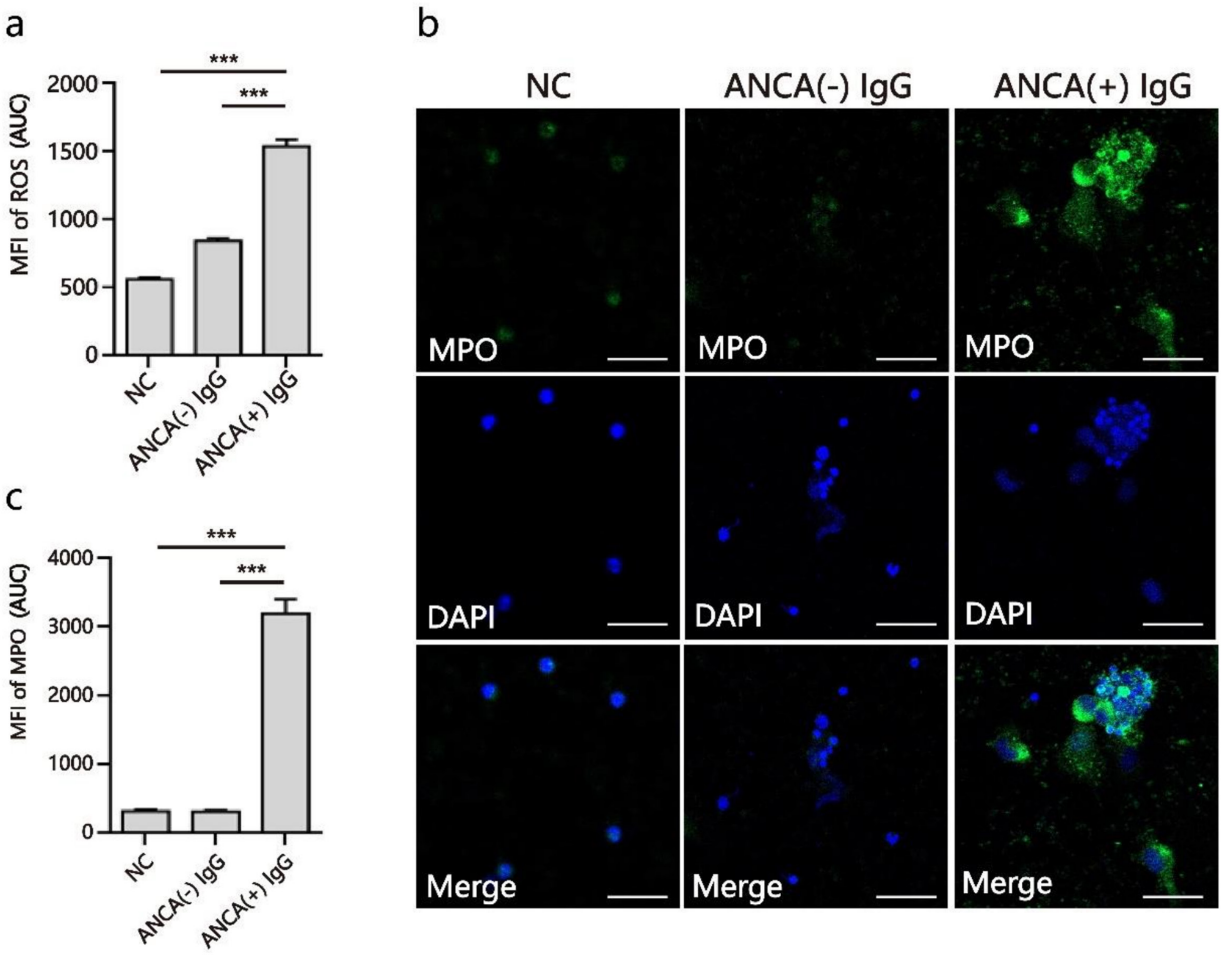
ROS and NETs induction in blood PMNs by ANCA treatment. PMNs were untreated or preincubated with IgG obtained from control patients with negative ANCA or BO patients with positive ANCA at 37°C for 24 h. (a) Analysis of mean fluorescence intensity (MFI) showed ROS generation in PMNs untreated or stimulated with IgG, in the presence or absence of ANCA. (b) Representative image of confocal immunofluorescence showed NETs release after PMNs untreated or stimulated with IgG in the presence or absence of ANCA, and immunofluorescent staining was conducted with anti‐MPO antibody (green) or DAPI (blue). Scale bars, 50 μm. (c) The MFI of MPO in ANCA (+) IgG‐treated PMNs compared with control groups was analyzed. Data are represented as mean ± SD of three independent replicates. ***p < 0.001. NC, negative control.

We previously reported marked ANCA accumulation in plasma from patients with BO correlated with BO disease severity [4, 5, 6]. To determine whether ANCA activated neutrophils to release NETs in the process of BO, human PMNs were pretreated with IgG obtained from BO patients with high ANCA expression at a concentration of 5 μg/mL for 24 h. We first examined ROS production by using a probe of DHR123 and detecting the fluorescence intensity. As seen in Figure 2a, the intracellular ROS released by ANCA‐positive IgG‐stimulated PMNs was significantly increased (*p* < 0.001). Additionally, immunofluorescence revealed robust PMN activation based on MPO staining by the treatment of ANCA‐positive IgG (Figure 2b), which produced a significantly higher level of NETs compared with the other two control groups (*p* < 0.001 for both; Figure 2c).

### 3.3 NETs‐Induced Pro‐Inflammatory Factors Production From Airway Epithelial Cells in a Dose‐Dependent Manner

**FIGURE 3:**
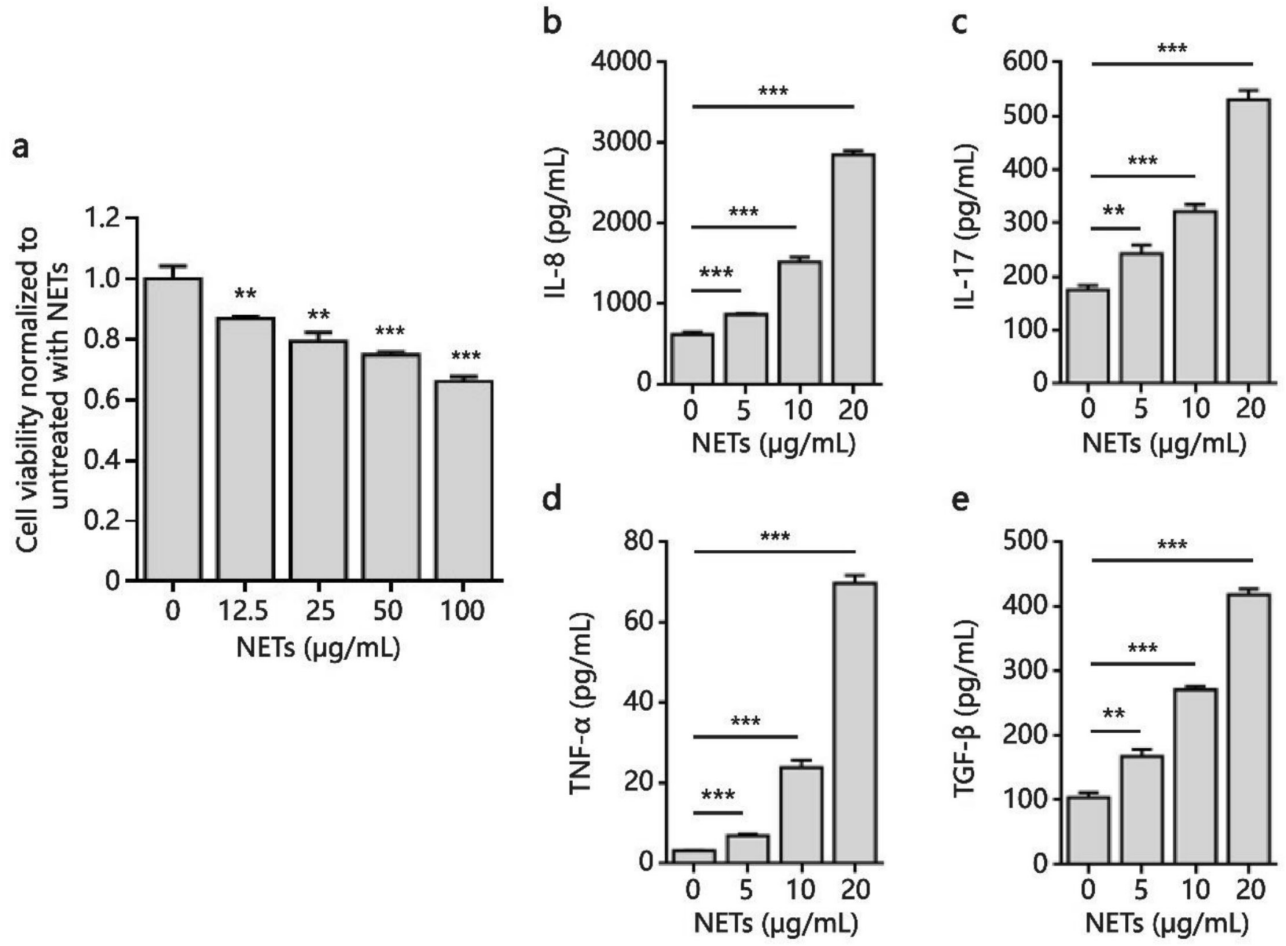
NETs induced cytokines production from SAECs. NETs were produced by peripheral blood PMNs treated with IgG obtained from BO patients with positive ANCA at 37°C for 24 h, then collected and quantified by PicoGreen assay. SAECs were treated with different concentrations of NETs for 24 h. A, (a) Reducing cell viability of SAECs by NETs as measured by CCK‐8 assay. The levels of IL‐8 (b), IL‐17 (c), TNF‐*α* (d), and TGF‐*β* (e) in culture supernatants were assessed by ELISA. Data are represented as mean ± SD of three independent replicates. **p < 0.01, ***p < 0.001 compared with control group without NETs treatment.

Knowing that NETs can induce cytotoxicity, we assessed the viability of SAECs after 24 h of treatment with NETs induced by ANCA IgG obtained from BO patients using the CCK‐8 assay. As shown in Figure 3a, the CCK‐8 analysis revealed that the cell viability of SAECs was significantly reduced upon treatment with NETs in a concentration‐dependent manner.

To demonstrate a direct relationship between NETs induced by ANCA and airway inflammation, we assessed whether NETs could induce pro‐inflammatory factor production in a monoculture of SAECs and detected a range of cytokines in the culture supernatants as indicators of airway epithelial inflammation. ELISA analysis showed that the levels of IL‐8 (Figure 3b), IL‐17 (Figure 3c), TNF‐*α* (Figure 3d), and TGF‐*β* (Figure 3e) were increased by treatment with NETs in a dose‐dependent and statistically significant manner compared to control cells.

### 3.4 Pro‐Inflammatory Effects of ANCA on Co‐Cultured Cells

**FIGURE 4:**
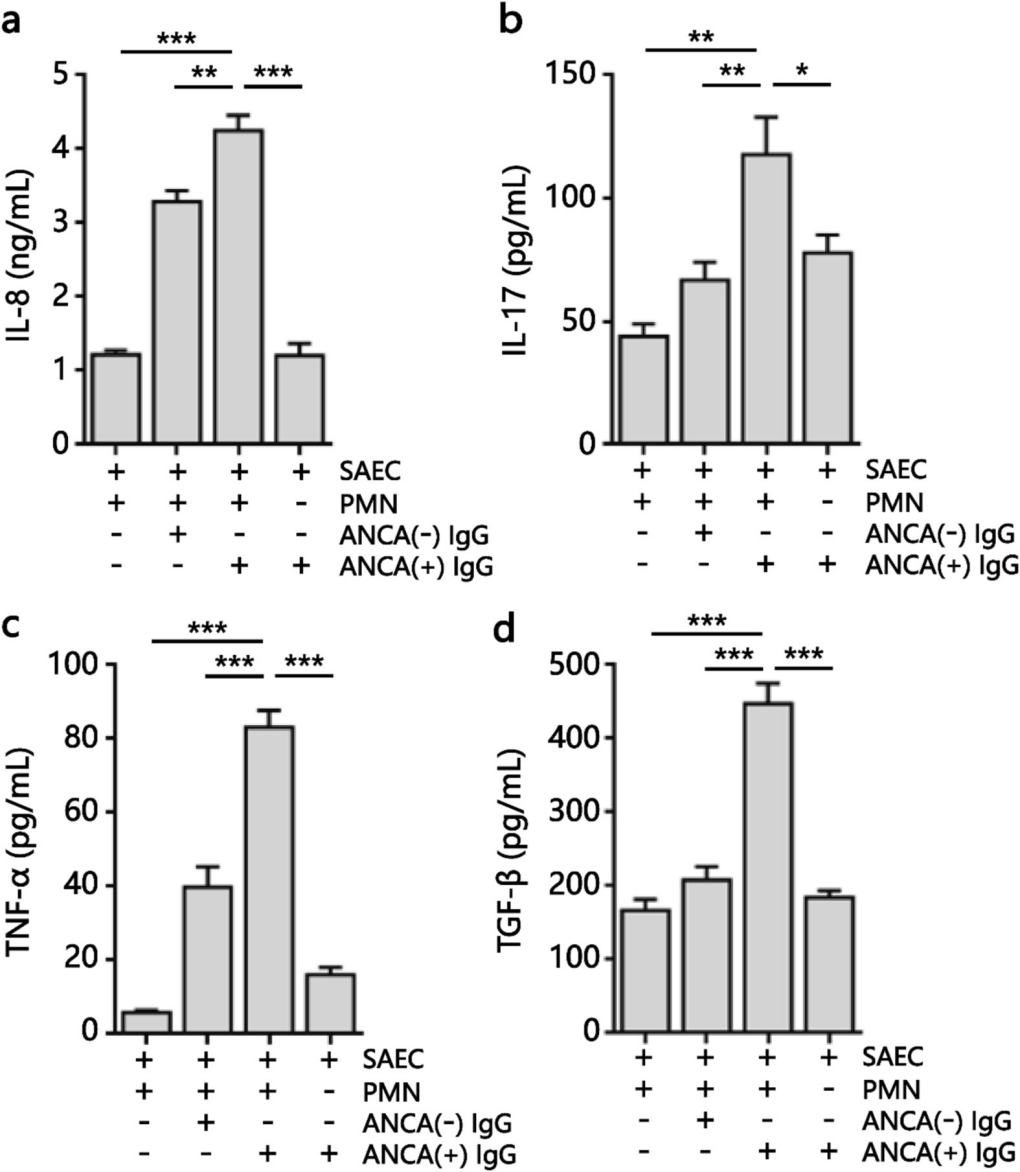
ANCA induced cytokines production from co‐cultured cells of PMNs and SAECs. We assessed production levels of IL‐8 (a), IL‐17 (b), TNF‐*α* (c), and TGF‐*β* (d) using ELISA from the supernatant of SAEC or co‐cultured cells treated with IgG obtained from control patients with negative ANCA or BO patients with positive ANCA at 37°C for 24 h. Data are represented as mean ± SD of three independent replicates. *p < 0.05, **p < 0.01, ***p < 0.001. SAEC, small airway epithelium cell. PMN, polymorphonuclear cell.

The pro‐inflammatory effects of ANCA were evaluated by measuring a range of cytokine production levels in co‐cultured cells from SAECs and PMNs treated with ANCA‐positive IgG. We found that the levels of IL‐8 (Figure 4a), IL‐17 (Figure 4b), TNF‐*α* (Figure 4c), and TGF‐*β* (Figure 4d) were significantly increased in the supernatant of co‐cultured cells treated with IgG obtained from BO patients with high ANCA expression. Interestingly, there were no effects on the cytokine production in the supernatant of SAECs treated with ANCA‐positive IgG, which indicated that the pro‐inflammatory function of ANCA was probably induced via the activation of PMNs.

## 4 Discussion

BO is a severe and refractory chronic lung disease caused by a variety of causes [1], among which severe lower respiratory tract infection is the most common cause, and more than 70% of children with BO are caused by severe pneumonia [7, 15]. Several studies have suggested that neutrophils are involved in the process of chronic airway injury and abnormal host repair, and the persistent neutrophilic inflammation in small airways plays a key role in the development of BO [16, 17]. Our previous study found for the first time that the autoantibody ANCA, which targets the cytoplasmic components of neutrophils, was persistently expressed abnormally in children with BO after infection [8]. Further clinical studies showed that the level of ANCA was closely related to the clinical severity of children [9].

Building upon our prior clinical findings, the present study mechanistically links elevated ANCA to sustained neutrophilic inflammation in BO airways. We demonstrate that circulating ANCA is not merely a biomarker but a functional driver of neutrophil activation, as evidenced by its ability to induce robust ROS and NETs production ex vivo. This connects the clinical observation of high ANCA titers to a specific pro‐inflammatory neutrophil phenotype [18, 19]. Previous studies on ANCA mostly focused on vasculitis, and ANCA is considered to be the hallmark antibody of ANCA‐associated vasculitis [3, 10]. In the classical pathway of vasculitis, ANCA binds to antigens on the membrane surface of neutrophils to activate the cells. On the one hand, neutrophils are induced to undergo respiratory burst and degranulation in a short time, releasing a large number of cytotoxic superoxide and proteolytic enzymes, which directly damage vascular endothelial cells [20]. On the other hand, ANCA promotes neutrophils to release NETs, which attach to vascular endothelial cells and cause apoptosis [21]. As an immune regulatory system, NETs not only have the ability to trap and kill pathogens but are also highly toxic to tissues and cells. Their over‐expression and clearance disorders can cause additional inflammatory damage to the host. Some researchers have found that NETs were highly expressed in pulmonary microvessels in animal models of ventilator‐induced lung injury and transfusion‐related acute lung injury [22, 23]. Veras et al. have also found that surviving SARS‐CoV‐2 can induce normal neutrophils to produce NETs and trigger the death of lung epithelial cells [24]. Collectively, these reports establish a precedent for NETs‐mediated damage in pulmonary pathology. Recent studies have further revealed that NETs activate NLRP3 inflammasomes in airway epithelial cells, amplifying inflammatory cascades [25], and that NETs‐derived proteases directly impair epithelial barrier integrity in cystic fibrosis [26]. In our preliminary investigation, western blot analysis of lung tissues from a murine BO model demonstrated NETs activation, characterized by significantly elevated expression of NE and MPO [27]. In line with this concept, our data extend the role of NETs to BO by demonstrating elevated NETs components in patient plasma. The elevation of these specific NETs components in patient plasma not only confirms in vivo but also suggests their potential as measurable indicators of disease activity.

We next sought to understand how ANCA and NETs might contribute to the inflammatory milieu in BO. Notably, the co‐culture of ANCA‐stimulated neutrophils with SAECs provoked a pronounced inflammatory response, characterized by the secretion of IL‐8, IL‐17, TNF‐*α*, and TGF‐*β*. To directly implicate NETs in this process, we demonstrated that purified NETs alone are sufficient to elicit a similar cytokine profile from SAECs in a dose‐dependent manner, thereby identifying NETs as a crucial mediator of ANCA's effects.

The specific cytokines upregulated in our experiments have profound implications for BO pathogenesis. Both IL‐8 and TNF‐*α* are classical neutrophil activators, which can induce respiratory burst and degranulation in a short time [28] and promote the release of NETs [29]. IL‐17 is an inflammatory cytokine that upregulates the expression of inflammatory genes, either by inducing de novo gene transcription or by stabilizing target mRNA transcripts [30]. TGF‐*β* is recognized as a key factor in airway epithelial fibrosis [31, 32]. Therefore, we propose a self‐amplifying vicious cycle central to BO progression: ANCA‐primed neutrophils release NETs, which damage the airway epithelium and provoke it to release IL‐8, TNF‐*α*, and TGF‐*β*. These cytokines can then recruit and activate further neutrophils, potentially leading to more ANCA production and NETs. This cycle perpetuates chronic inflammation and drives fibrotic remodeling, offering a mechanistic explanation for the relentless nature of BO. Despite these limitations, our study provides initial evidence linking ANCA and NETs to BO pathogenesis.

In conclusion, our findings suggest that the ANCA‐NETs pathway contributes to airway inflammation in children with BO. Future studies are needed to validate this model in vivo, dissect the signaling pathways involved, and explore the therapeutic potential of targeting this axis.

## Author Contributions

Xiaowen Chen and Shangzhi Wu performed the collection of data, drafted the article, and edited the manuscript. Zhenwei Liu, Zhanhang Huang and Jiaxing Xu performed the collection of data and revised the manuscript. Zhongji Wu, Hui Li and Hongwei Li performed the experiments in vitro. Dehui Chen conceptualized and designed the study, performed data analysis, and revised the manuscript. The manuscript has been read and approved for submission by all authors. All authors approve this version to be published.

## Funding

This work was supported by the General Guidance Project of Guangzhou Health Science and Technology [Grant number 20251A011067], the Science and Technology Program of Guangzhou [Grant number 202102010276], the National Natural Science Foundation of China [Grant number 81770063], and the Results and Clinical Transformation Project of the First Affiliated Hospital of Guangzhou Medical University [Grant number ZH202111].

## Ethics Statement

The study was conducted in accordance with the Declaration of Helsinki (as revised in 2013). The study was approved by the Ethics Committee of the First Affiliated Hospital of Guangzhou Medical University (reference number: ES‐2023‐011‐01).

## Consent

Written informed consent to be included in the study was obtained from the parents of the included children.

## Conflicts of Interest

The authors declare no conflicts of interest.

## Data Availability

The datasets used and/or analyzed during the current study are available from the corresponding author on reasonable request.
